# Phytochemical analysis, antimicrobial and radical-scavenging properties of *Acalypha manniana* leaves

**DOI:** 10.1186/2193-1801-2-503

**Published:** 2013-10-03

**Authors:** Jaures AK Noumedem, Jean de Dieu Tamokou, Gerald Ngo Teke, Rosine CD Momo, Victor Kuete, Jules Roger Kuiate

**Affiliations:** Department of Biochemistry, Faculty of science, University of Dschang, P.O. Box 67, Dschang, Cameroon; Department of Medicine, Faculty of Health Sciences, University of Bamenda, P.O. Box 39, Bambili, Cameroon; Laboratory of Microbiology and Antimicrobial Substances, Faculty of Science, University of Dschang, P.O. Box 67, Dschang, Cameroon

**Keywords:** *Acalypha manniana*, Euphorbiaceae, Extracts, Phytochemicals, Antimicrobials, Free radical scavenging

## Abstract

*Acalypha manniana* (Euphorbiaceae) is a plant popularly used in Cameroon and in several parts of Africa for the treatment of various microbial diseases like diarrhea and skin infections.

The present study was designed to evaluate the phytochemical composition, antimicrobial and radical-scavenging activities of *A. manniana* methanol leaf extract and its fractions*.*

The methanol extract was partitioned into hexane, ethyl acetate and residual fractions and phytochemical analysis was conducted using standard methods. The broth microdilution method was used to evaluate the antimicrobial activity against nine bacterial species and four dermatophyte species. The free radical scavenging activities of the methanol extract and its fractions were evaluated using the 2,2-diphenyl-1-picrylhydrazyl (DPPH) assay.

The results obtained showed that *A. manniana* contains alkaloids, tannins, anthocyanins, flavonoids, phenols and steroids. The methanol extract as well as the hexane, ethyl acetate and residual fractions exhibited both antibacterial and antidermatophytic activities that varied between the microbial species (MIC = 0.12 – 2.04 mg/mL). These tested samples also showed high radical-scavenging activities (RaS_50_ = 3.34 – 4.80 μg/mL) when compared with vitamin C used as reference antioxidant (RaS_50_ = 1.74 μg/mL).

These findings provide evidence that the studied plant possesses antimicrobial and antioxidant properties and may act as potential antioxidant for biological systems susceptible to free radical-mediated reactions.

## Background

The search for new antimicrobial and antioxidant agents from natural sources has intensified in response to the limitations of currently available therapy and the emergence of drug-resistant strains. Plants produce diverse range of bioactive molecules, making them a rich source of different types of potential drugs (Walton 1999; Ghasemzadeh et al. 2010; Jaiswal et al. 2010). Herbal drugs have gained importance in recent years because of their efficacy and cost effectiveness (Ripa et al. 2009). Today, nearly 88% of the global populations turn to plant derived medicines as their first line of action for maintaining health and combating diseases (Govindarajan et al. 2008). According to the World Health Organization, medicinal plants would be the best source to obtain a variety of drugs (Nascimento et al. 2000). Thus, many plants that are of medicinal importance have been investigated by various researchers (Ali et al. 2000; Prasad et al. 2004; Kuete et al. 2008; Souza et al. 2008; Tenjoh et al. 2012). The success story of chemotherapy lies in the continuous search for new drugs to counter the challenge posed by resistant strains of microorganisms (Haruna et al. 2013). The investigation of certain indigenous plants for their antioxidant and antimicrobial properties may yield useful results. Cameroon has a great variety of natural vegetation, which is used in traditional medicine to cure various ailments. Among the plants used for medicinal purpose in Africa, particularly in Cameroon is *Acalypha manniana*.

*Acalypha manniana* Müll. Arg., belonging to the family of Euphobiaceae, is a slender climbing shrub that grows to about 6 m high in marshy places. According to some traditional healers found in the Western region of Cameroon, decoction of the leaves is used for the treatment of mycosis and skin diseases. A leafy stem decoction of *A. manniana* is taken in some African countries like Ivory Coast, Ghana, Uganda, Rwanda, Burundi and Cameroon to treat diarrhoea (Schmelzer 2007; Shmelzer et al. 2008). Previous studies have reported the presence of phenolics, tannins, alkaloids, steroids, flavonoids, glycosides and saponins in the leaf extracts of *A. alnifolia* (Evanjelene & Natarajan 2012) and *A. indica* (Mohan et al. 2012). The ethanolic extract of *A. wilkesiana* leaves revealed the presence of tannins, steroids, flavonoids and cardiac glycosides while saponins, alkaloids and anthraquinones were not present (Gotep et al. 2010). A variety of phenolic compounds and fatty acids methyl ester were found in the *A. monostachya* extracts (Canales et al. 2011). The antifungal properties of extracts of leaves of *A. hispida* have been established (Ejechi & Souzey 1999). Gallic acid, corilagin and geraniin have been reported to be the active compounds responsible for the antimicrobial activity of *A. wilkesiana* while quercetin 3-O-rutinoside and kaempferol 3-O-rutinoside were also isolated from the inactive fraction of *A. hispida* (Adesina et al. 2000). The antibacterial, antifungal and/or antioxidant activities of *A. monostachya*, *A. wilkesiana, A. indica, A.alnifolia*, *A. hispida* were also mentioned (Alade & Irobi 1993; Canales et al. 2011; Shirwaikar et al. 2004; Govindarajan et al. 2008; Durga et al. 2009; Onocha et al. 2011; Evanjelene & Natarajan 2012; Haruna et al. 2013). However, no systematic work has been undertaken on analyzing the extracts of *A. manniana.* In the continuation of the strategy of new drug discovery, we studied the phytochemical composition, antimicrobial and antioxidant activities of the *A. manniana* methanol leaf extract and its three derivative fractions.

## Materials and methods

### Plant material

The leaves of *Acalypha manniana* were collected in Dschang, West Region of Cameroon, in February 2009. Authentification of the plant was done at the Cameroon National Herbarium, where the voucher specimen was deposited under the accessory number 18223/SRF/CAM.

### Preparation of the methanol extract and its fractions

The collected leaves were washed before being air-dried for three weeks. The dried leaves were ground into fine texture using an electric blender. The air-dried and powdered leaves (1 Kg) were extracted by maceration in six liters of methanol for 72 hours at room temperature. Thereafter, it was filtered with Whatman paper number one and the methanol evaporated at 50°C under reduced pressure using a rotary evaporator (Buchi Rotavapor R-200). This yielded 212 g of concentrated extract. A portion of 60 g of methanol extract was successively partitioned with *n*-hexane and ethyl acetate to yield 5.54 g of hexane fraction, 3.70 g of ethyl acetate fraction, and 48.62 g of residual fraction. The methanol crude extract and its fractions were stored at +4°C till usage for phytochemical and biological assays.

### Phytochemical screening

Phytochemical analysis for antimicrobial and antioxidant compounds was done using standard methods described by (Bruneton 1999) for alkaloids, anthocyanins, flavonoids, phenols, triterpenes, steroids, saponins, tannins, anthraquinones and coumarins.

### Microorganisms and culture media

The microorganisms used in this study consisted of two Gram-positive bacteria (*Staphylococcus aureus* ATCC25922, *Enterococcus faecalis* ATCC10541), three Gram-negative bacteria (*Escherichia coli* ATCC11775, *Pseudomonas aeruginosa* ATCC27853, *Klebsiella pneumoniae* ATCC13883) which are reference strains obtained from the American Type Culture Collection, and four clinical isolates (*Proteus mirabilis*, *Salmonella paratyphi A*, *Salmonella paratyphi B*, *Shigella flexneri*) kindly provided by the Pasteur Centre (Yaoundé-Cameroon). Three dermatophyte strains (*Trichophyton mentagrophytes* E1425, *Trichophyton terrestre* E1501, *Microsporum gypseum* E1420) were collected from “Ecole Nationale Vétérinaire d’Alford, France” and one clinical isolate (*Trichophyton equinum*) kindly provided by the Pasteur Centre (Yaoundé-Cameroon). The bacterial and dermatophyte strains were grown at 37°C and 28°C on nutrient agar (NA, Conda, Madrid, Spain) and Sabouraud Dextrose Agar (SDA, Conda) slants respectively (Tamokou et al. 2009).

### Preparation of microbial inoculum

The inocula of bacteria were prepared from 24 h old broth cultures. The absorbance was read at 600 nm and adjusted with sterile physiological solution to match that of a 0.5 McFarland standard solution. From the prepared microbial solutions, other dilutions with sterile physiological solution were prepared to give a final concentration of 10^6^ colony-forming units (CFU) per milliliter (Tamokou et al. 2012).

Conidia suspensions of dermatophyte species were prepared from 10 days old cultures respectively. The number of conidia was determined using a spectrophotometer and adjusted with sterile saline (NaCl) solution (0.90%) to an absorbance of 0.600 at 450 nm corresponding to a final concentration of about 1×10^5^ spores/mL (Venugopal & Venugopal 1992).

### Antimicrobial assay

The minimum inhibitory concentrations (MICs), minimum bactericidal concentrations (MBC) and minimum fungicidal concentrations (MFCs) were determined as previously described (Tamokou et al. 2009; Fogue et al. 2012)

For every experiment, a sterility check (5% aqueous DMSO and medium), negative control (5% aqueous DMSO, medium and inoculum) and positive control (5% aqueous DMSO, medium, inoculum and water-soluble antibiotics) were included. MICs were assessed visually after the corresponding incubation period and were taken as the lowest sample concentration at which there was no growth or virtually no growth. The assay was repeated thrice.

For the minimum microbicidal concentration (MMC) determination, 10 μL aliquots from each well that showed no growth of microorganism were plated on Mueller-Hinton Agar or Sabouraud Dextrose Agar and incubated at 37°C for 24 h (bacteria) and at 28°C for 10 days (dermatophytes). The lowest concentration that yielded no growth after the sub-culturing was taken as the MBCs or MFCs. Gentamycin (for bacteria) and griseofulvin (for dermatophytes) were used as positive controls.

### Antioxidant assay

The free radical scavenging activity of the crude extract and fractions was evaluated as described by Tamokou et al. (2012) with slight modifications. The test samples were prepared in methanol and 100 μL of each sample added to 900 μL of 2,2-diphenyl-1-picryl-hydrazyl-hydrate ( 20 mg/L DPPH) methanol solution, to give final concentrations of 50, 100, 200, 400 and 800 μg/mL. L-ascorbic acid was used as a positive control and 1mL of 20 mg/L DPPH methanol solution was used as negative control. The content of each preparation was mixed and incubated at room temperature in a dark cupboard. The absorbance was then monitored after 30 min and converted into percentage of scavenging activity:

The experiments were carried out in triplicate and the percentages of DPPH scavenged by test samples were compared to that of L-ascorbic acid. These radical scavenging percentages were plotted against the logarithmic values of the concentrations and a linear regression curve was established in order to calculate the RSa_50_, which are the amounts of sample necessary to decrease by 50% the free radical DPPH.

### Statistical analysis

Statistical analysis was carried out using Statistical Package for Social Science (SPSS, version 12.0). The experimental results were expressed as the mean ± Standard Deviation (SD). Group comparisons were performed using One Way ANOVA followed by Waller-Duncan Post Hoc test. A p value of 0.05 was considered statistically significant.

## Results and discussion

The result from the present investigation showed the presence of alkaloids, phenols, flavonoids, anthraquinones, anthocyanins, tannins and steroids in the *A. manniana* leaf extract (Table 1). These compounds varied within the fractions. In agreement with our results, the phytochemical screening of the leaf extracts of *Acalypha hispida, A. marginata, A. racemosa, A. monostachya, A. alnifolia*, *A. wilkesiana* and *A. indica* revealed the presence of phenolics, tannins, steroids, flavonoids, glycosides, saponins and anthraquinones that varied within the plant species (Iniaghe et al. 2009; Gotep et al. 2010; Canales et al. 2011; Evanjelene & Natarajan 2012; Mohan et al. 2012).

**Table 1:** **Main groups of potential antimicrobial and antioxidant compounds found in the extracts of** ***A. manniana***

	Extraction Yield (g/100g)	Physical aspect	Main groups of antimicrobial/antioxidant compounds									
			Alca	Antho	Anthra	Couma	Flavo	Phen	Sapo	Ster	Tan	Triter
**Methanol extract**	11.50	Dark green sticky	+	+	+	-	+	+	-	+	+	-
**Hexane fraction**	9.23	Dark creamy	-	+	-	-	-	+	-	+	-	-
**Ethyl acetate fraction**	6.16	Green streaky	+	+	-	-	+	+	-	+	+	-
**Residual fraction**	81.03	Brown powdery	+	+	+	-	-	+	-	-	+	-

- : absent; + : present; Alca: alkaloids; Antho: anthocyanins; Anthra: anthraquinones; Couma: coumarins; Flavor: flavonoids; Phen: phenols; Sapo: saponins; Ster: steroids; Tan: tannins; Triter: triterpens.

The results in Table 2 show that the methanol extract and its fractions prevented the growth of all tested microorganisms, with MIC values ranging between 0.12 and 2.04 mg/mL for bacteria, and from 0.25 to 1.02 mg/mL for dermatophytes. *S. flexneri*, *P. mirabilis* and *T. equinum* (MIC= 0.25 - 0.51 mg/mL) were the most sensitive microorganisms while *S. aureus* (MIC= 0.25 – 2.04 mg/mL) was the most resistant. The lowest MIC value for these tested samples (MIC = 0.12 mg/mL) was obtained with ethyl acetate fraction on *P. aeruginosa*. The findings of the present study explain why the plant extracts are used in traditional folk medicine and confirm the activity of *A. manniana* as a broad spectrum antimicrobial agent since it inhibited the growth of Gram-positive (*S. aureus*, *E. faecalis*) and gram negative bacteria (*E. coli*, *P. aeruginosa*, *K. pneumoniae, P. mirabilis*, *S. paratyphi A*, *S. paratyphi B*, *S. flexneri)* as well as some fungi (*T. mentagrophytes*, *T. terrestre*, *T. equinum*, *M. gypseum*). Similar findings had been reported for other species by Canales et al. (2011) and Haruna et al. (2013). The antimicrobial activities of *A. manniana* (MIC = 0.25 - 2.04 mg/mL) can also be considered very important compared with those of Haruna et al. (2013) who reported the antimicrobial activities of the leaves of *A. wilkesiana* methanolic extract and its four derivative fractions (MIC = 25–100 mg/mL) on human pathogenic bacteria namely *S. aureus*, *S. pyogenes*, *E. faecalis*, *P. aeruginosa*, *P. vulgaris*, *E. coli, Aspergillus niger*, *A. flavus*, *A. carbonerium*, *T. mentagrophytes* and *Candida albicans*. The fact that the methanolic extract of *A. manniana* and its fractions showed activity against most of the test microorganisms has added another plant in bank of herbal medicines.

**Table 2:** **Inhibition parameters (MIC, MBC) of methanol extract and its fractions from** ***A. manniana leaves*** **(mg/mL)**

	***Microorganisms***	Parameters	Methanol extract	Hexane fraction	Ethyl acetate fraction	Residual fraction	Ref*
***Bacteria***	***Salmonella typhi***	**MIC**	0.25	2.04	0.51	0.25	0.032
		**MBC**	4.09	8.19	4.09	1.02	0.12
		**MBC/MIC**	8	4	8	4	4
	***Staphylococcus aureus***	**MIC**	0.25	2.04	0.51	2.04	0.002
		**MBC**	4.09	8.19	4.09	8.19	0.016
		**MBC/MIC**	8	4	8	2	8
	***Pseudomonas aeruginosa***	**MIC**	0.51	1.02	0.12	2.04	0.002
		**MBC**	2.04	4.09	2.04	4.09	0.016
		**MBC/MIC**	4	4	8	2	8
	***Escherichia coli***	**MIC**	0.51	1.02	0.51	0.25	0.016
		**MBC**	2.04	4.09	1.02	2.04	0.064
		**MBC/MIC**	4	4	2	8	4
	***Klebsiella pneumoniae***	**MIC**	**0.51**	1.02	0.51	0.51	0.008
		**MBC**	4.09	4.09	4.09	4.09	0.016
		**MBC/MIC**	8	4	8	8	2
	***Shigella flexneri***	**MIC**	0.25	0.51	0.51	0.51	0.002
		**MBC**	1.02	1.02	1.02	4.09	0.002
		**MBC/MIC**	4	2	2	16	1
	***Enterococcus faecalis***	**MIC**	0.25	1.02	0.51	1.02	0.001
		**MBC**	1.02	2.04	1.02	2.04	0.001
		**MBC/MIC**	4	2	2	2	1
	***Proteus mirabilis***	**MIC**	0.51	0.51	0.51	0.51	0.001
		**MBC**	0.51	2.04	1.02	4.09	0.001
		**MBC/MIC**	1	4	2	8	1
*Dermatophytes*	***Trichophyton mentagrophytes***	**MIC**	0.25	1.02	0.51	0.51	0.01
	***T. equinum***	**MIC**	0.25	0.25	0.25	0.51	0.005
	***T. terrestre***	**MIC**	0.51	0.51	0.25	1.02	0.005
	***Microsporum gypseum***	**MIC**	0.25	0.25	0.51	1.02	0.01

*Ref = reference drugs: gentamycin for bacteria and griseofulvin for dermatophytes.

Our data showed that the responses of microorganisms to tested substances varied in term of sensitivity among the strains. The differences in susceptibility may be explained by the differences in cell wall composition and/or in genetic content of plasmids that can be easily transferred among microbial strains (Karaman et al. 2003). It may also be explained by differences in the mechanism by which the active principles of the plant extracts exert their effect (Takeo et al. 2004). It was also observed that the ratios MBC/MIC found for the tested substances were generally less than or equal to 4 (Table 2), on the corresponding microbial species, suggesting that the killing effects of the extract/fractions could be expected on most of the tested micro-organisms (Tene et al. 2008; Tamokou et al. 2009). This is very interesting in view of the perspective of new antibacterial discovery from this plant, when considering the medical importance of the tested microorganisms. Although the MIC values were ranging between 0.25 and 1.02 mg/mL for the dermatophytes, the methanol extract and fractions exerted fungistatic effects, suggesting the formation of resistance structure of dermatophytes at high concentrations (Georgopoulos & Skylakakis 1986).

The results of the antioxidant activities of the methanol extract, hexane, ethyl acetate and residual fractions are presented in Table 3. It appeared that the methanol extract and its fractions displayed important radical-scavenging activities against DPPH (RaS_50_ = 3.34 – 4.80 μg/mL) when compared with L-ascorbic acid used as reference antioxidant (RaS_50_ = 1.74 μg/mL). These observations demonstrate that the tested samples are free radical inhibitors or scavengers acting possibly as primary antioxidants. This is very promising in the perspective of new antioxidant discovery from plant extracts. The antimicrobial activity was more concentrated in the methanol extract and contrarily, the antioxidant activity is more concentrated in the residual fraction. This suggests that *A. manniana* contains several antioxidant and/or antimicrobial principles with different polarities as shown by the phytochemical analysis. The residual fraction (Rsa50 = 3.34 μg/mL) was more active than the methanol extract (Rsa50 = 4.51 μg/mL) and hexane fractions (Rsa50 = 4.81 μg/mL) indicating that the active principles might be more concentrated in residual fraction and more diluted in others tested samples. The free radical scavenging activities of *A. manniana* (RSa_50_ = 3.34 – 4.80 μg/mL) are highest compared with those of Evanjelene & Natarajan (2012) who reported the percentages of scavenging of the aqueous (91.56%), chloroform (52.55%), petroleum ether (48.34%) and methanol (88.76%) extracts of *A.alnifolia* leaves on DPPH at 0.16 mL of extracts per milliliter of solution.

**Table 3:** **DPPH radical scavenging activity of the methanol extract and its fractions from** ***Acalypha manniana*** **leaves**

Test substances	RSa_50_(μg/mL)
Methanol extract	4.51 ± 1.28^c^
Hexane fraction	4.80 ± 0.01^c^
Ethyl acetate fraction	4.01 ± 0.07^bc^
Residual fraction	3.34 ± 0.00^b^
L-ascorbic acid	1.74 ± 0.01^a^

RSa_**50**_ **:** concentration of tested sample necessary to decrease by 50% the free radical DPPH. Values bearing the different superscript letters are significantly different (p< 0.05).

Many plant extracts exhibit efficient antioxidant properties due to their phytoconstituents, including phenolics and nitrogenous compounds (Durga et al. 2009; Evanjelene & Natarajan 2012; Tatsimo et al. 2012; Tamokou et al. 2013), which were found to be present in our extracts. DPPH is a stable free radical at room temperature and accepts an electron or hydrogen radical to become stable diamagnetic molecules (Soares et al. 1997). Phenolics and nitrogenous compounds are known to be potential antioxidant due to their ability to scavenge free radicals through donation of hydrogen, forming the reduced form of DPPH. Therefore, the presence of such compounds may be responsible for the antioxidant activity found in the methanol extract and its fractions.

## Conclusions

The methanol extract of *A. manniana* showed the highest antimicrobial activity while the residual fraction displayed the largest scavenging activity against DPPH confirming the traditional use of this plant in the treatment of various bacterial diseases such as diarrhea and skin infections. The residual fraction of *A. manniana* extract can be explored for its applications in the prevention of free radical related diseases. The obtained results might be considered sufficient for further studies geared towards the isolation and identification of the active principles and to evaluate possible synergistic effects among the extract components for their antimicrobial and radical-scavenging properties. Toxicity studies will be also necessary to establish if the crude extract/fractions could be safely used as antimicrobial/antioxidant agents.
